# Ethnical Disparities in Response to Edaravone in Patients With Amyotrophic Lateral Sclerosis

**DOI:** 10.7759/cureus.25960

**Published:** 2022-06-15

**Authors:** Maleesha Jayasinghe, Rahul Jena, Malay Singhal, Samiksha Jain, Snigdha Karnakoti, Minollie Suzanne Silva, Abdul Mueez Alam Kayani

**Affiliations:** 1 Medicine, Nanjing Medical University, Nanjing, CHN; 2 Medicine, Bharati Vidyapeeth Medical College, Bharati Hospital, Pune, IND; 3 Internal Medicine, Mahatma Gandhi Memorial Medical College, Indore, IND; 4 Medicine, Guntur Medical College, Guntur, IND; 5 Medicine, Malla Reddy Institute of Medical Sciences, Hyderabad, IND; 6 Medicine and Surgery, Nanjing Medical University, Nanjing, CHN; 7 Medicine and Surgery, Allama Iqbal Medical College, Lahore, PAK

**Keywords:** neurodegenerative disease, motor neuron disease, motor neurons, neurodegenerative disorders, genetics, ethnicity, riluzole, amyotrophic lateral sclerosis, edaravone, als

## Abstract

Amyotrophic lateral sclerosis (ALS), also known as motor neuron disease (MND), is a progressive neurodegenerative disease characterized by the weakness of voluntary muscles due to the loss of motor neurons. Symptoms ultimately culminate in the form of respiratory failure due to the involvement of the diaphragm. Unfortunately, there is no known cure for this disease. Hence, supportive therapy is the only available option in most terminal cases. However, Riluzole and Edaravone (EDA) are the only two known drugs approved by the U.S. Food and Drug Administration (FDA) for treating this condition. In 2017, EDA was approved for the treatment of ALS. It is hypothesized that Riluzole and EDA work via a mechanism involving antioxidants, which nullifies the oxidative stress believed to be involved in ALS. However, most studies in several countries have found a wide range of disparities in the efficacy of this drug. In this review, we aim to summarize the differences in results from epidemiological studies across 10 different countries and hypothesize the potential causes of these differences.

## Introduction and background

Amyotrophic lateral sclerosis (ALS), also known as Charcot's disease or Lou Gehrig's disease, is a progressive neurodegenerative disorder characterized by the degeneration of motor neurons in the brain and spinal cord. Within two to five years, ALS naturally progresses to muscle atrophy, paralysis, and death due to respiratory insufficiency [1]. Approximately 90% of ALS cases are sporadic, caused by genetic load, aging, and environmental factors in susceptible people. The remaining cases are familial and associated with numerous genetic mutations, including C9ORF72, superoxide dismutase 1 (SOD1), TAR DNA-binding protein, fused in sarcoma, and ubiquilin-2 mutations [2]. Various pathogenic processes, such as misfolded protein aggregation within cells, glutamate excitotoxicity, neuroinflammation, oxidative stress, and defective energy metabolism, are believed to contribute to ALS [3]. ALS is diagnosed by clinical examination and electrodiagnostic tests, including nerve conduction studies and electromyography, which are used to diagnose ALS. In the case of discrepancies between clinical presentation, medical history, and instrumental findings, neuroimaging or serological studies, such as cerebrospinal fluid analysis, can be performed to rule out ALS [4]. Several qualitative scales, such as the ALS Functional Rating Scale-Revised (ALSFRS-R), ALSFRS-Extension (ALSFRS-EX), ALS Milano-Torino Staging (ALS-MITOS), CNS-Bulbar Function Scale (CNS-BFS), Dyspnea-ALS-Scale (DALS-15), Motor Neuron Disease-Dyspnea Scale (MND-DS), and Rasch-Built Overall ALS Disability Scale (ROADS), have been identified that can be used to assess the functional status of ALS patients [5]. The revised El Escorial criteria and the Awaji-Shima criteria are used to stratify ALS patients into subgroups in large randomized clinical trials [4]. Two drugs currently approved for the treatment of ALS are edaravone (EDA), a free radical scavenger, and riluzole, a glutamate antagonist, despite their limited effects on disease progression.

In 2017, the United States Food and Drug Administration (FDA) approved EDA for the treatment of ALS. EDA, also known as 3-methyl-1-phenyl-2-pyrazoline-5-one, is a substituted 2-pyrazoline-5-one derivative. It comes in intravenous and oral formulations [6]. In 1987, Mitsubishi Chemical Industries (which includes Mitsubishi Yuka Pharmaceutical Corporation; Tokyo, Japan) began clinical trials on EDA under the code name MCI-186. In 2001, Mitsubishi Tokyo Pharmaceutical Inc. initiated clinical trials of EDA for ALS. EDA was designated an orphan drug (a pharmacological agent produced to treat extremely rare medical disorders that would be unprofitable to produce without government funding is known as an orphan drug) in 2005 for the treatment of ALS. In 2015, the Japanese government granted commercial approval for the use of EDA to treat ALS. In 2017, the United States FDA approved EDA [7].

**Figure 1:**
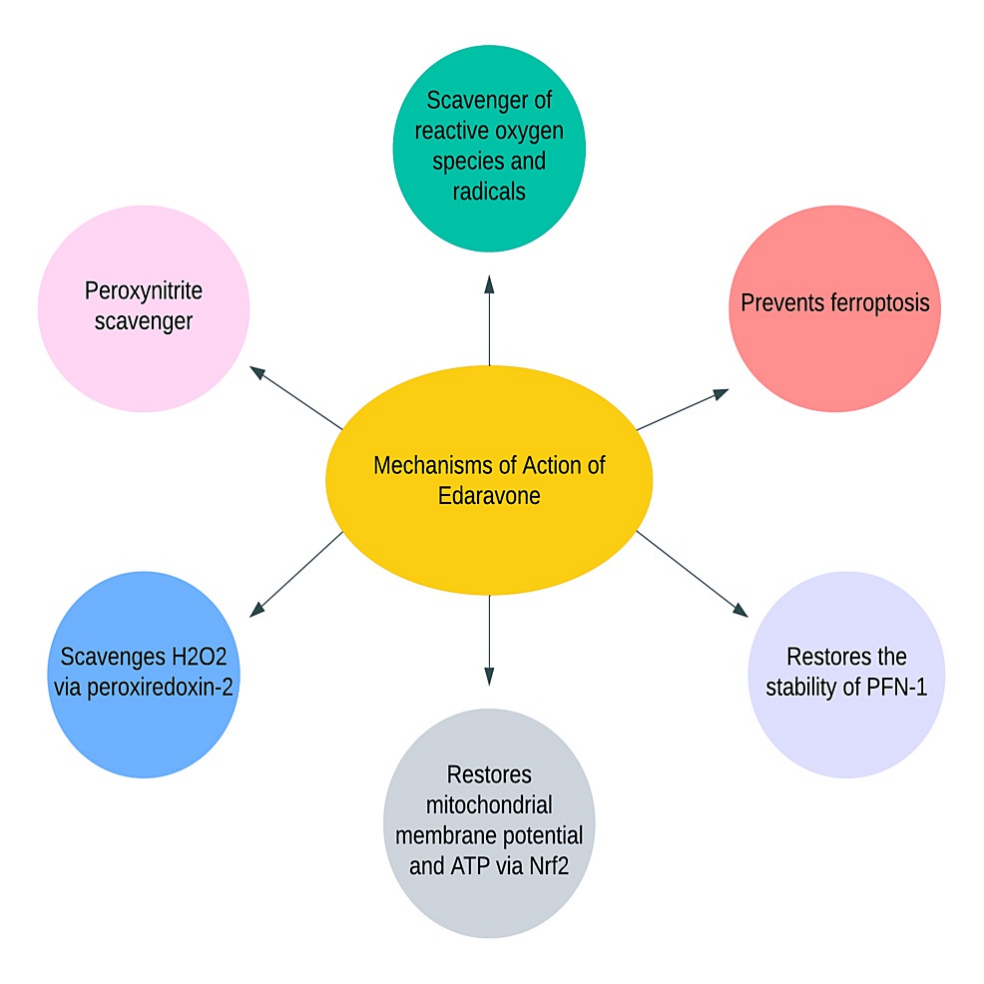
Mechanisms of Action of EDA H2O2: hydrogen peroxide; ATP: adenosine triphosphate; Nrf2: nuclear factor-erythroid factor 2-related factor 2; PFN-1: profilin-1; EDA: edaravone Original figure, made by author Maleesha Jayasinghe

Although the precise mechanism of action of EDA is unknown, its therapeutic effect may be attributable to its antioxidant properties. The likely mechanisms of EDA are depicted in Figure 1.

Although EDA is one of only two FDA-approved drugs for the treatment of ALS, the results of multiple studies conducted in different parts of the world have been inconclusive and inconsistent. After EDA administration, there has been a significant reduction in the ALSFRS-R and forty-item ALS assessment questionnaire (ALSAQ-40) scores and significant preservation of gross motor function [8]. However, the drug's effect on the life expectancy of ALS patients has not yet been determined.

Some patients who meet stringent selection criteria, such as those used in the MCI-186-J19/NCT01492686 study, like being older than 18 years, a decrease in the ALSFRS-R score of one to four during a 12-week period between screening and baseline, disease duration no longer than two years, a score of two or more in all of the items of ALSFR-R, ‘definitive’ or ‘probable’ ALS according to the El Escorial criteria, and a forced vital capacity (FVC) of 80% [9], have shown encouraging responses to the drug in preliminary studies. Given the heterogeneity of disease progression and other factors, it is difficult to identify patients who precisely meet this criterion in real-world clinical practice. As a result, additional clinical studies are needed to establish a broader criterion for patients who would benefit from the medication.

Age, psychological factors, nutritional status, comorbidities, and the amount of time between the onset of the disease and the administration of EDA must all be considered when interpreting results in ALS patients. There is often a delay in the diagnosis of ALS from the time the disease first manifests itself. Finding novel tests and techniques (new criteria) to diagnose the disease at a younger age would allow us to begin treatment at an earlier stage of the disease, thereby increasing the likelihood of a positive outcome for patients.

In the course of analyzing the results of numerous global studies, we found that studies conducted in Japan and Korea demonstrated a statistically significant treatment effect while studies conducted in Europe, India, and Kuwait did not. This may be the result of regional variations in genomic architecture. It is crucial to evaluate the potential genetic differences involved in the pathogenesis of ALS and their effect on the therapeutic potential of EDA. ALS has been linked to a number of genes that contribute significantly to its inheritance and susceptibility. SOD1 mutations have been linked to the antioxidant properties and ability to reduce the oxidative stress of EDA. A case report from China identified a Cu/Zn SOD1 gene mutation as the cause of ALS, making it a case of familial ALS [10]. Understanding the function of this gene is crucial because it provides us with numerous benefits. Prenatal genetic screening and prophylactic treatment of asymptomatic individuals with mutations in this gene could substantially enhance their quality of life [10]. In addition, evaluating genetic variations, mutations, and certain racial preponderances may aid in determining differences in response to EDA. Incorporating the results of genetic analysis as one of the inclusion criteria for EDA-treated patients may help us identify trends between ethnicity and genetics and response to EDA. In addition, conducting global studies would enable us to identify the role of genetics in EDA's effects more precisely and help us achieve better results in the future.

Using data from studies conducted in different countries, this study aims to assess the efficacy of EDA and attempts to explore the potential effect of ethnicity on this drug's efficacy.

## Review

Methods

Included in the searched databases were PubMed, PubMed Central (PMC), ResearchGate, and Google Scholar. These were extensively searched using relevant keywords and Medical Subject Heading (MeSH) terms to identify all relevant articles on the use of EDA in the treatment of amyotrophic lateral sclerosis. Both MeSH terms "Edaravone" and "ALS" were included in the search. We included articles from 2015 to 2022 and included reviews, animal studies, observational studies, and clinical trials. We only included studies for which full-text articles were available. Additionally, only studies published in English were considered.

Results

An advanced MeSH search on PubMed and Google Scholar yielded 3521 articles at first. All papers not directly related to the research topic were manually omitted, leaving 27 studies for our review.

Discussion

The following are the findings of studies conducted in 10 different countries on the use of EDA in patients with ALS.

Germany

One-hundred ninety-four patients with ALS initiated intravenous EDA treatment (141 patients received more than or equal to four treatment cycles; 130 patients were matched), and 130 patients whose propensity scores were matched received standard therapy. All patients with disease onset between December 2012 and April 2019 who met the EI Escorial criteria for clinically probable or definite ALS were included in the study. Subgroups were defined by applying the inclusion criteria of the MCI186-ALS19 study [9] to determine whether patients were eligible (EFAS) or ineligible (non-EFAS). The primary outcome was measured by a decline in the ALSFRS-R score. The secondary outcomes were the likelihood of survival, the time to ventilation, and the progression of the disease before and during treatment. Sixteen percent of patients exhibited potential adverse effects. The progression of the disease did not differ between 116 patients treated with EDA for a median of 13.9 months and 116 patients treated with standard therapy for a median of 11.2 months. The probability of survival, time to ventilation, and progression of disease were not significantly different. Similarly, there was no difference in the outcomes for EFAS subgroups and non-EFAS subgroups between EDA-treated and matched patients [11].

Japan

In a study conducted in Japan between 2011 and 2014, 137 patients who met all of the following criteria (two or more points on each of the 12 items of the ALSFRS-R, 80% FVC, definite or probable ALS, and two-year disease duration) were randomly assigned to one of two treatment groups. One arm of the study received 60 mg of intravenous EDA for six cycles while the other arm received a placebo for six cycles. The participants were then given the option to continue with the 24-week open-label extension. One-hundred twenty-three patients completed the 48-week extension period: 65 in the EDA-EDA (E-E) group and 58 in the placebo-EDA (P-E) group. In the E-E group, the ALSFRS-R score changed almost linearly from Cycle 1 to Cycle 12. However, there was no sudden decline in the score [12].

Houzen et al. conducted a study on 45 ALS patients hospitalized between 2013 and 2018. Twenty-two patients were treated with EDA for 26.6 months while the remaining patients served as the control group. Tracheostomy, positive-pressure ventilation, or death were the key outcomes, and the study's follow-up period ended in 2020. Kaplan-Meier analysis revealed that the survival rate in the EDA group was significantly higher than in the control group, with a median survival of 49 months in the EDA group and 25 months in the control group. Overall, the findings of this single-center retrospective study suggest that EDA may prolong the lives of patients with ALS [13].

India

A single-center observational study was conducted on ALS patients over the age of 18 who met the El Escorial criteria for possible, probable, or confirmed ALS. They were given EDA for 24 weeks (6 cycles). In Cycle 1, the drug was administered for two weeks, followed by a drug-free period of two weeks. In Cycles 2 and beyond, the study drug was administered for the first 10 days, followed by a drug-free interval of 18 days. The primary efficacy endpoint was defined as at least a 20% improvement in ALSFRS-R score relative to baseline. The secondary outcome measure was a 10% increase in jitter. Thirty patients were enrolled in the study, and 23 of them successfully completed treatment. Eighty percent of patients had limb atrophy, while 93.3% reported limb weakness. Fasciculations at various endpoints lacked statistical significance. The single-fiber electromyography (EMG) jitter differences of the patients improved marginally, but not statistically significantly [14].

Italy

An observational study of patients treated with EDA in 39 ALS centers was conducted between May 2017 and May 2019. All patients enrolled in the study met the MCI-186-J19 [9] inclusion criteria. The reduction of ALSFRS-R and FVC in ALS patients treated with at least 12 months of edaravone was compared to a group of matched controls from the Pooled Resource Open-Access ALS Clinical Trials database using descriptive and survival analytic methods. This study recruited a total of 331 patients treated with EDA and 290 matched historical controls. In both descriptive and survival analyses, there were no significant changes in disease progression or respiratory function between the two cohorts [15].

Korea

From 2016 to 2018, 22 ALS patients who received EDA treatment were identified. All patients with ALS were diagnosed using the El Escorial criteria. ALSFRS-R was evaluated from the initial treatment through six cycles of treatment. Pulmonary function was evaluated using FVC and forced expiratory volume in one second. The patients were categorized as limb-onset or bulbar-onset. A 60-minute infusion of EDA was administered intravenously once daily. According to the standard protocol, EDA was administered for two weeks, followed by a two-week drug-free period, and then for 10 days within two weeks. Eighteen of the enrolled patients had a limb-onset type while four had a bulbar-onset type. Nineteen patients met the diagnostic criteria for definitive ALS while three patients had probable ALS. Fifteen patients who had received riluzole in the past continued to receive it. Two patients out of a total of 22 met the inclusion criteria for the second phase three trial: two points on each of the 12 items of the ALSFRS-R, 80% FVC, revised El Escorial criteria for probable or definite ALS, and a disease duration of two years. Six patients dropped out. Sixteen patients ultimately completed six treatment cycles. Twelve of the patients had the limb-onset type while four patients had the bulbar-onset type. The ALSFRS-R and pulmonary function tests showed moderate improvement in ALS patients treated with EDA [16].

USA

A survey of 67 physicians in the United States revealed that 3,007 patients were prescribed EDA, with 67% of patients using it in conjunction with riluzole, 24% having used riluzole at some point, and 9% being riluzole naive. Fifty-nine percent of the patients who received the drug had an implanted port, 21% used a peripherally inserted central catheter, and 18% utilized a peripheral line. Forty-three percent of patients were administered EDA at home while the remaining patients were administered the medication in clinics. More than 50 cases of drug inefficacy were reported [17]. Differences in methods of administration and discrepancies in administering riluzole may have affected the outcomes.

Kuwait

The study included 17 patients with ALS (12 with limb onset and five with bulbar onset) who met the MCI-186-J19 study criteria [9]. This study was a prospective cohort observational study that assessed participants at 24, 28, and 72 weeks after EDA infusion. The primary outcomes of the study were the rates of ALSFRS-R and FVC decline. Other factors evaluated included the necessity for non-invasive ventilation, mechanical ventilation, ambulation, tube feeding, tracheostomy tube, drug satisfaction, and survival rate. In the study, riluzole or other ALS treatments were permitted, and there was no comparison group. There was a statistically significant decline in ALSFRS-R at 72 weeks and in the Medical Research Council score at 48 and 72 weeks but not in FVC. The study found that after one year of EDA therapy, there was a significant decline in function with preserved respiratory function. The drug did not meet expectations in this study [18].

Iran

Twenty patients who met the following inclusion criteria were included in the study: revised El Escorial criteria for probable or definite ALS, mild to moderate disease (according to the ALS Health State Scale), scored two points on all items of the Revised ALSFRS-R, and had an FVC of at least 80%. One arm (consisting of 10 patients) received EDA while the other arm (consisting of 10 patients) served as the control. Twelve cycles were observed between the two arms (each cycle lasted four weeks). The treatment group was administered EDA for the first 14 days of the first cycle and the first 10 days of subsequent cycles. In addition, riluzole was administered to each patient. Every three cycles, the ALSFRS-R, ALSAQ-40, and Manual Muscle Testing (MMT) scores were measured to assess the physical and functional status of the patients. The ALSAQ-40, ALSFRS-R, and MMT scores did not differ significantly between the treated and control groups at any of the five-time points. The study failed to demonstrate the effectiveness of EDA in treating ALS [19].

Argentina

The study describes its application to 16 patients (14 limb onset and two bulbar onset) between 2016 and April 2020. During the research, the characteristics of EDA-treated patients were analyzed. There were no criteria for patient selection. Except for one patient, all others received riluzole at the same time. The median time between ALS diagnosis and EDA infusion treatment was 19.8 months. One patient experienced thrombophlebitis while another experienced limb pain. Aside from these side effects, the majority of patients exhibited good tolerance to the drug. Two patients discontinued treatment due to dissatisfaction. One of the four patients who underwent tracheostomy died. This study was limited by the fact that insurance companies do not cover edaravone in Argentina, which acted as a limitation [20]. If insurance covered the drug, more people would have used it, and they would have been able to determine whether the drug produced satisfactory results. The absence of criteria of selection and the presence of one patient, even though not very statistically significant, are other important considerations in this study.

Israel

In this study, 22 patients were treated with EDA, and 71 patients who were not treated with EDA were found to be effective without using the initial clinical trials' specific criteria. For at least six months, the majority of patients had been taking a stable dose of riluzole. In the study, two groups of ALS patients were compared: those treated with EDA and those not treated with EDA. The rate of decline of the ALSFRS-R, MMT, and time to death or tracheostomy were compared between the two groups. None of the parameter comparisons revealed a statistically significant difference. The mortality rates of patients who received treatment were higher, but this difference was not statistically significant. Within hours of receiving infusions, one-third of patients experienced respiratory complications; these patients had lower FVC scores at baseline. It is essential to note that none of the patients satisfied the criteria for a positive clinical trial for EDA [21]. This could be a significant factor affecting the outcomes of the patients.

We noticed conflicting results in studies conducted across different countries. All the observational studies and clinical trials included in our study are summarized in Table 1.

**Table 1:** A compilation of studies on the response of ALS patients to EDA undertaken in various countries ALSFRS-R: Amyotrophic Lateral Sclerosis Functional Rating Scale-Revised; EDA: Edaravone; ALS: Amyotrophic Lateral Sclerosis; FVC: Forced Vital Capacity; ALSAQ-40: Forty-Item ALS Assessment Questionnaire; MMT: Manual Muscle Testing

Author	Country of study	Year	Study design	Key result	Conclusion	Study weakness
Witzel et al. [11].	Germany	2017 to 2020	Cohort	Not significant	There were no differences in survival, time to ventilation, or disease progression (ALSFRS-R score) between patients treated with EDA and those treated with standard therapy.	
WRITING GROUP ON BEHALF OF THE EDARAVONE (MCI-186) ALS 19 STUDY GROUP [12].	Japan	2011 to 2014	Open-label	Significant	From cycles 1 to 12, the ALSFRS-R score changed almost linearly. The percent forced vital capacity, Modified Norris scale score, or ALSAQ-40 all remained unchanged.	During the active treatment extension period, there was no statistical test or placebo group.
Houzen et al. [13].	Japan	2013 to 2018	Retrospective Study	Significant	EDA improved survival and sustained its effect over a number of years. Minor adverse effects of long-term EDA treatment were noted.	Small number of patients
Tomar et al. [14].	India	(Published in 2022)	Observational study	Not significant	Patients experienced adverse effects including limb atrophy and fasciculations. There was no statistically significant difference between ALSFRS-R scores at various endpoints.	Small number of patients
Lunetta et al. [15].	Italy	2017 to 2019	Observational study	Not significant	There were no significant differences between EDA-treated and untreated ALS patients in terms of disease progression (ALSFRS-R score) or respiratory function (FVC).	-
Park et al. [16].	Korea	2016 to 2018	Observational study	Significant	The ALSFRS-R score and pulmonary function improved marginally. There were reports of mild adverse effects such as eczema and pruritus.	Small number of patients
Ortiz et al. [17].	United States	2018	Blinded survey	-	153 cases of drug inefficacy and several other adverse effects were reported.	Small number of patients
Ismail et al. [18].	Kuwait	2018 to 2020	Observational study	Not significant	Despite preserved respiratory function (FVC) and a high safety profile, there was a significant functional decline (ALSFRS-R score) after one year of EDA therapy.	-
Eishi-Oskouei et al. [19].	Iran	2017 to 2019	Parallel-group, single-blinded clinical trial	Not significant	At 5 different time points, there were no significant differences in disease progression (ALSAQ-40, ALSFRS-R, or MMT scores) between cases and controls. There were no injection reactions or significant differences in side effects between cases and controls.	-
Quarracino et al. [20].	Argentina	2016 to 2020	Retrospective study	-	The EDA treatment delay was five times greater than the riluzole treatment delay. Adverse reactions were rare. EDA was well-tolerated but treatment access was limited.	Small number of patients, no inclusion criteria, and no insurance coverage for drug
Abraham et al. [21].	Israel	2017-2018	Retrospective study	Not significant	The rate of monthly decline of ALSFRS-R was not different between EDA treated and untreated patients in our real-life study. EDA was not effective in unselected ALS patients.	A small number of patients, insufficient data retrieval, unselected patients, and a short diagnostic delay among treated patients.

The disparities in outcomes observed with the use of EDA in patients with ALS could be attributed to genetics, the uncertain mechanism of action of EDA, and the unclear pathophysiology of ALS. More research is needed to better understand these elements of both the drug and the disease to discover a clear connection between the effects of EDA in ALS patients and achieve the best survival outcomes with EDA.

Limitations

There is a significant disparity among the studies in various countries, which may be explained by genetic differences. However, a huge gap exists in this regard in terms of research. Hence, there is a need for cross-sectional studies and trials to deal with this gap. This review is limited to studies in the English language, so we may have missed valuable studies published in other languages. In addition, the studies conducted before 2015 are excluded, which may also have caused a similar limitation.

## Conclusions

EDA is believed to act in ALS and other neurodegenerative diseases via a wide spectrum of mechanisms. These range from membrane stabilization to free radical scavenging. Our review found that all European countries, India, and Kuwait conducted studies where EDA failed to live up to its expectations while the studies conducted in the Southeast Asian countries (except India) had significant positive effects, however mild. As noted previously, there is a huge discrepancy among the results of various countries when it comes to the efficacy of EDA. In order to know why it is essential to consider the role of genetics. Additionally, we need to consider the effects of other factors, such as food, formulations, and gender, as well. At present, we have very limited knowledge about the basics of the disease, right from the pathophysiology of ALS to how the drug works. Hence, the need of the hour is to conduct larger-scale studies.
